# PW03-026 - Caspase-1 variants involved in ER stress

**DOI:** 10.1186/1546-0096-11-S1-A252

**Published:** 2013-11-08

**Authors:** H Luksch, V Schlipfenbacher, S Winkler, J Roesler, A Rösen-Wolff

**Affiliations:** 1 Department of Pediatrics, University Hospital Carl Gustav Carus, Dresden, Germany

## Introduction

Caspase-1 is a proinflammatory enzyme that is activated by the NLRP3 inflammasome in response to endoplasmic reticulum (ER) stress independent of the classical unfolded protein response. This finding linked ER stress to chronic inflammatory diseases. In patients suffering from unexplained recurrent febrile episodes we detected several genetic variants of *CASP1* leading to reduced enzymatic activity due to destabilization of the caspase-1 dimer interface.

## Objectives

We investigated a possible association of reduced enzymatic activity of variant caspase-1 with impaired ER-stress responses.

## Methods

We analyzed ER stress markers in THP-1 cells and lymphoblastoid cells lines (LCL, EBV transformed B cells) from healthy donors and individuals with *CASP1* variants. Additionally, we knocked down endogenous caspase-1 in THP-1 cells to analyze caspase-1 involvement in ER stress responses. We used quantitative real time RT-PCR to examine mRNA expression of genes involved in ER stress and Western blot detection of Bip (GRP78). Quantification of IL-1β, IL-8, and TNF-α secretion was performed by cytometric bead arrays.

## Results

As expected, expression levels of spliced Xbp1, Bip, EDEM, and Chop were increased after induction of ER stress by Tunicamycin (inhibitor of protein glycosylation) in THP-1 cells and LCLs. In THP-1 cells such induction of ER stress lead to secretion of IL-1β, IL-8, and TNF-α. On the other hand, LPS induced activation of the NLRP3 inflammasome and increased expression of ER stress related genes. Furthermore, spliced XBP1 and Bip expression were significantly increased in unstimulated *CASP1* knock-down THP-1 cells and in native patients’ LCLs expressing variant caspase-1 with reduced enzymatic activity.

## Conclusion

These data indicate that ER stress induced activation of wildtype caspase-1 might be involved in a negative feed back reduction of ER stress and that impaired caspase-1 activity might allow for a perpetuation of ER stress thus contributing to the proinflammatory phenotype of our patients.

## Disclosure of interest

None declared

